# 

**Published:** 1912-09

**Authors:** 


PUBLISHED MONTHLY. ATLANTA SUBSCRIPTION $1.00

### ATLANTA JOURNAL-RECORD OF MEDICINE

Successor to { Atlanta Medical and Surgical Journal, Established 1855. and Southern Medical Record, Established 1870. } 57th Year

Vol. LIX. ATLANTA, GA., SEPTEMBER, 1912. No. 6

